# Perspective: Practical Approach to Preventing Subclinical B12 Deficiency in Elderly Population

**DOI:** 10.3390/nu13061913

**Published:** 2021-06-02

**Authors:** Alessandra Vincenti, Laura Bertuzzo, Antonio Limitone, Giuseppe D’Antona, Hellas Cena

**Affiliations:** 1 Laboratory of Dietetics and Clinical Nutrition, Department of Public Health, Experimental and Forensic Medicine, University of Pavia, 27100 Pavia, Italy; hellas.cena@unipv.it; 2 Glaxosmithkline (GSK) Consumer Healthcare, via Zambeletti s.n.c., 20021 Baranzate, Italy; laura.x.bertuzzo@gsk.com (L.B.); antonio.x.limitone@gsk.com (A.L.); 3 Centro di Ricerca Interdipartimentale nelle Attività Motorie e Sportive (CRIAMS)—Sport Medicine Centre, University of Pavia, 27058 Voghera, Italy; giuseppe.dantona@unipv.it; 4 Clinical Nutrition and Dietetics Service, Unit of Internal Medicine and Endocrinology, ICS Maugeri IRCCS, 27100 Pavia, Italy

**Keywords:** Vitamin B12, cobalamin, elderly, micronutrient deficiency, hidden hunger, prevention, sustainability

## Abstract

Vitamin B12 (also known as cobalamin) is an essential water-soluble vitamin that plays a pivotal role for several physiologic functions during one’s lifespan. Only certain microorganisms are able to synthetize B12, thus humans obtain cobalamin exclusively from their diet, specifically from animal-derived foods. Specific sub-group populations are at risk of vitamin B12 subclinical deficiency due to different factors including poor intake of animal source foods and age-dependent decrease in the capacity of intestinal B12 uptake. Consumption of animal products produces some negative health issues and negatively impacts sustainability while a plant-based diet increases the risk of B12 deficiency. Taking a cue from the aforementioned considerations, this narrative review aims to summarize facts about B12 deficiency and the burden of inadequate dietary intake in elderly population, as well as to discuss sustainable approaches to vitamin B12 deficiency in aging population.

## 1. Introduction

Adequate intake of nutrients is essential to maintain overall health through lifespan. Growing evidence has shown that (sub)clinical nutrient inadequacies as well as deficiencies affect health and quality of life [1]. It is well known that vitamin B12 (B12) deficiency is common in specific sub-groups including the elderly [2]. Data from the National Health and Nutrition Examination Survey (NHANES) show in 6.9% and 15% prevalence of B12 deficiency in US adults respectively aged 51–70 and over 70 years [3].

Vitamin B12 is an essential water-soluble vitamin [4], also known as cobalamin, which contains ion cobalt in its structure. It is mainly found in animal products, especially meat, seafood, eggs, milk and dairy products [5]. Only microorganisms (certain archaea and bacteria [5] are able to synthetize cobalamin, thus humans obtain B12 exclusively from their diet, specifically from animal products. Plant-origin foods do not contain B12, except for algae and fortified food items (e.g., cereals, fortified substitutes of milk, flours, etc.) [6].

**Figure 1:**
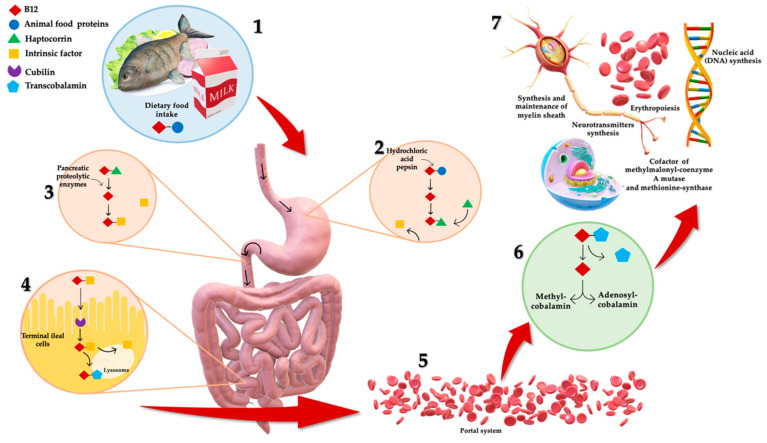
Complex mechanism of B12 absorption, metabolism and functions. (1) Dietary B12 is obtained through animal foods. (2) B12 release takes place in the stomach by means of hydrochloric acid and pepsin. Here, it is bounded to haptocorrin, forming a protein-complex. (3) Once arrived in duodenum, B12 is released from its protein-complex due to pancreatic proteolytic enzymes. Free B12 is then bound by intrinsic factor (IF). (4) B12–IF complex reaches terminal ileum where it is absorbed. Afterward, the complex is degraded in lysosomes and B12 is bound to transcobalamin, forming transcobalamin–B12 complex. (5) B12 is transported via the portal system in this complexed form, (6) and it is uptaken and accumulated by body cells, where it is converted to metabolic active forms: Methylcobalamin and Adenosylcobalamin. (7) B12 is crucial for several physiologic functions: erythropoiesis, synthesis and maintenance of myelin sheath, DNA and neurotransmitters synthesis, and intracytoplasmic cofactor.

B12 absorption and metabolism are quite complex (as shown in Figure 1), and a number of physiological factors contribute to clinical manifestation of deficiency, frequently observed in the elderly [4]. Dietary B12 release takes place in the stomach by means of hydrochloric acid and pepsin [4]. Here, vitamin B12 is bounded to R-protein, named haptocorrin, secreted by salivary glands and stomach [4]. Once arrived in duodenum, B12 is released from its protein-complex due to pancreatic proteolytic enzymes (i.e., trypsin, chymotrypsin and elastase) [2]. Free B12 is then bound by intrinsic factor (IF), a glycoprotein secreted by parietal cells in the mucosa of the stomach [4]. B12–IF complex remains in this form until it reaches the terminal ileum where it is absorbed through endocytosis [4]. Afterward, the complex is degraded in lysosomes and vitamin B12 is eventually bound to transcobalamin (TC), forming TC-Cbl complex [2]. B12 is transported via the portal system in this complexed form [2], and it is uptaken and accumulated by body cells, where it is converted into the metabolic active forms, Methylcobalamin and Adenosylcobalamin [4].

Vitamin B12 is crucial for several physiologic functions, including erythropoiesis, synthesis and maintenance of myelin sheath, as well as synthesis of nucleic acid (DNA) and neurotransmitters synthesis [7,8]. It is also involved in intracytoplasmic biochemical pathways as pivotal cofactor for two enzymes: (i) the mitochondrial methylmalonyl-coenzyme A (CoA) mutase, and (ii) the cytosolic methionine-synthase [4]. The first one is involved in propionate metabolism catalyzing isomerization of methylmalonyl-CoA to succinyl-CoA; the second one is involved with other B-vitamins in the cytosolic transmethylation of homocysteine (Hcy) to methionine by 5-methyl-tetrahydrofolate [4]. During B12 deficiency, substrates of both B12-dependent reactions are accumulated, leading to increased levels of Hcy and methylmalonic acid (MMA) in plasma [2]. Excessive Hcy in blood (hyperhomocysteinemia) is associated with several chronic diseases including cardiovascular and neurodegenerative diseases, peripheral neuropathy, renal failure and hypothyroidism [9]. Hyperhomocysteinemia has also been associated to sarcopenia (defined as an age-related loss of muscle mass and function) and could mediate inhibition of the satellite cell proliferation (resident muscle precursor/stem cells with regenerative capacities properties) [10]. The latter effect is mediated by (i) enhancing the p38 MAPK signalling in different tissue types; (ii) enhancing the oxidative damage in skeletal muscles; and (iii) by inducing myostatin, an inhibitor of myogenesis, in skeletal muscles [11]. 

Increase in circulating MMA level is associated with an overall acidification of the body and defective fatty acid synthesis of neuronal membranes [2]. 

Vitamin B12 deficiency develops insidiously over the years, affecting health state. The preclinical stage of deficiency, named sub-clinical deficiency, exhibits only non-specific symptoms [12] and often remains underdiagnosed with negative health impact in vulnerable groups, especially older adults [13]. The most frequent and evident clinical manifestations of B12 deficiency are megaloblastic anemia and neurological alterations (e.g., sensory and motor disturbances, particularly in the lower extremities, ataxia) [14]. Moreover, neurological disorders can occur in the absence of hematological manifestations [2]. B12 deficiency in the elderly is also associated with cognitive decline, dementia and psychiatric disorders, covering suicidal behaviors, psychosis and mania, intense agitation [15]. Indeed, Morris M. et al. reported cognitive decline in 549 community-dwelling individuals, mean age 74.8 ± 4.6 years, having low B12 serum levels (between 187–256.8 pmol/L) [16]. 

Low B12 levels have also been associated with increased inflammation, oxidative stress and increased susceptibility to infections. Vitamin B12 plays an important role in gut microbioma modulation [17], which in turn impacts on the development and function of both innate and adaptive immune system [18]. Furthermore, it improves high CD4/CD8 ratio and suppresses natural killer cells [19]. 

There is no a consensus concerning B12 reference intervals/cut-off values; however, the recommended cut-off for its deficiency is <148 pmol/L (200 pg/mL) of plasma or serum concentration [4]. 

The overall long-term strategy for controlling B12 deficiency is to promote consumption of foods rich in Vitamin B12, mainly animal products [2]. Nevertheless, the elderly rarely achieve the goal due to numerous impediments including socioeconomic (cost of food) and physiological ones (i.e., swallowing and/or chewing problems), low food access (residents of food deserts), poor diets or diets lacking animal products (e.g., vegetarian and/or vegan diets), as well as ecological, religious and cultural reasons [20]. Moreover, people are driven to consume less meat by greater awareness of the negative impact on the planet besides their own health, compensated by increasing proportion of fruits, nuts and legumes [21,22,23] and consequently by increasing the risk of vitamin B12 deficiency [23].

The need to promote healthy dietary patterns [21,24,25], mainly plant-based, raises the demand to identify complementary and more sustainable sources of B12, as an alternative to animal products and to implement public health programs, to design sustainable nutritional solutions.

Based on the previous considerations, the aim of this narrative review is to describe the prevalence of B12 deficiency and the burden of its inadequate intake in the elderly, addressing the need of sustainable, public health preventive approaches to face subclinical deficiency. 

## 2. Prevalence of Deficiency

The average B12 content of the body is estimated to be approximately 2–3 mg in healthy adults (range: 1–6 mg), mainly stored in the liver [4], although significant amounts of B12 are also found in the kidneys [4]. In plasma B12 is bound to transcobalamin (TC) (~10–30%) forming holotranscobalamin (HoloTC), corresponding to the biologically active form of the vitamin [26]. The major residual fraction of plasma cobalamin (~70–90%) is attached to haptocorrins (HCs), representing inert form of the vitamin [4]. Despite the large original reserves of B12, its deficiency is a rather frequent phenomenon, especially during aging [27].

It is known that B12 levels tend to decline with age, but there are conflicting data about the real prevalence of deficiency in this population due to limitations, including: (i) the vast differences among subjects included in epidemiological studies, (ii) studies conducted in different age rages and ethnicity, (iii) food consumption (e.g., fortified food or not), (iv) presence or absence of morbidities, (v) absence of a gold standard test for measurement, and (vi) different biomarkers and cut-off levels considered by different authors [20,28]. Indeed, many studies considered only serum B12 levels alone (with different cut-offs), while others used vitamin B12 combination with additional serum biomarkers, like Hcy and/or MMA [27]. Different technical platforms for these measurements additionally complicate comparisons.

It is well known that the prevalence of B12 deficiency increases with age, but notably varies among countries and ethnicity [29]. Data from the NHANES III (Third National Health and Nutrition Examination Survey) showed that 4.4% of US adults (*n* = 3450, aged >50 years) have low serum B12 concentration (<148 pmol/L) [30], with the lowest values reported by Wright JD. et al. 1998 in the non-Hispanic white population, followed by Mexican Americans, and the highest ones in the non-Hispanic blacks [31]. In studies from individual countries across Europe, the reported data are different depending on the country. Mild B12 deficiency (B12 concentrations <260 pmol/L plus plasma MMA > 0.32 μmol/L) was reported in 24% of healthy, free-living, older Dutch subjects (*n* = 105; age range 74–80 years) [32], while data from the Hordaland study reported serum B12 < 200 pmol/L in 5.9% of Norwegian participants (*n* = 1935; age range 71–74 years) [33]. In Latin America, 27% of adults resulted B12 deficient (<103 pmol/L) [34], while in China 7.2% of women and 12.0% of men sampled for a nutrition and health survey (*n* = 1350, aged 65 to 90 years) showed low serum vitamin B12 concentration (<258 pmol/L) [35]. Vitamin B12 deficiency (B12 < 150 pmol/L) resulted much higher in India, reaching 46% prevalence in Indian adults (*n* = 2014, aged 27–55 years) [36].

The estimated prevalence of Vitamin B12 deficiency also differs depending on the presence of morbidity in the elderly, ranging from 4–5% in community-living elderly [37,38] to about 30–40% in institutionalized subjects with multiple comorbidities [24], reaching rates around 23–35% in the individuals over 80 years [2] as confirmed by Meziere A et al. [39] and higher rated in elderly males rather than women [27].

## 3. B12 Deficiency: Dietary Intake and Bioavailability

A typical Western diet provides around 5–30 μg daily of B12, 1–5 μg of which is absorbed in the last part of the small intestine [40,41]. This amount is higher than both the Adequate Intake (AI) of B12, set at 4.0 μg for adults by the European Food Safety Authority (EFSA) (elderly included) [4] and the Recommended Dietary Allowance (RDA) set at 2.4 μg for U.S. adults by the Food and Nutrition Board [42]. Therefore, vitamin B12 deficit is rarely attributable to pure nutritional deficiency, even in the elderly [38], in western countries [43]. This hypothesis is supported by the data analysis based on nine dietary surveys conducted in Europe (Germany, Denmark, Portugal, Spain, Sweden and the UK) on 28015 adults and elderly, showing inadequate intake below 10% in elderly population (age >64 years) [44]. 

Economic and social factors also play a role on vitamin B12 intake first of all due to limited affordability of animal source foods (ASFs) [45]. Several studies have been conducted in low- and middle-income countries, highlighting this important association, especially in infants and children [46,47,48]. Moreover, Mark HE. et al. [49], by means of the National Food Balance Sheet 2009, which provided information on the ability of the national food supply to ensure adequate fulfillment of nutrient requirements on a population level, estimated the prevalence of micronutrient inadequacies, including vitamin A, thiamine, riboflavin, folate, vitamin B-12, zinc and calcium, in seven low-middle income countries in the South Asia regions [49]. 

Apart from unavailability of ASFs, inadequate intake may also be driven by religious, cultural or personal reasons (e.g., vegan diets, or less frequently, restrictive vegetarian diets [7,50]). Moreover, B12 deficiency was observed in elderly with chewing and/or swallowing impairment, restricted mobility and/or immobility, depression and social isolation [51]. All these factors are frequently associated with malnutrition [52]. 

Another important aspect to be considered in the elderly is food insecurity which impacts on food choices [53] and contributes to over- and under-nutrition, nutrient excesses and deficiencies [54,55]. These factors affects both low-income countries as well as high income ones [56]. Food insecurity is inversely associated with higher levels of diet quality [57], which encompasses adequacy, moderation, variety or diversity, as well as balanced nutrition and food consumption [58]. A study based on 4009 elderly adults aged 74 or more found that food insecurity was associated with a poor diet quality [59]. For instance, it can lead to a “substitution” effect [60] where nutrient-dense foods, such as lean sources of protein, are replaced with energy-dense, nutrient-poor foods, usually ultra-processed ones rich in refined carbohydrates and fats [61], giving inadequate intake of B12. 

The maintenance of an optimal B12 status does not depend only on adequate dietary intake but also on B12 bioavailability in food [62]. Watanabe et al., in a study on 2861 subjects aged 71–74 years, concluded that milk and dairy products and fish were significant contributors to plasma B12, in fact vitamin B12 bioavailability from dairy products was higher than from other animal products [63]. The authors reported also a lack of association between meat and plasma B12 levels, raising doubts that cooking might contribute to significant losses (~33%) of B12, while milk and its products are largely consumed raw, safeguarding B12 bioavailability [64]. Another factor that needs to be considered is impact of heat processing of food on vitamin B12 bioavailability. Nishioka M. et al. demonstrated that B12 content of round herring meats decreased down to 59% (grilled for 7.5 min), to 47% (boiled for 5 min), to 41% (fried for 4 min), to 43% (steamed for 9 min) and to 59% (microwaved for 1 min) [65]. Evidence shows that B12 losses depend also on cooking temperature and time of cooking [65]. Heat processing of milk causes appreciable B12 losses, up to 30% and 50% when milk is boiled for 2–5 min and 30 min, respectively, 50% losses when microwaved for 5 min and 5–10% losses when pasteurized [66,67]. Those data suggested that changes in Vitamin B12 bioavailability due to time and cooking processes of food need to be considered in addition to the absolute amount of vitamin B12 content in food, when assessing dietary B12 intake.

Concerning dairy products, milk is likely to be the most important component of dairy intake impacting on serum B12 concentrations [62], in accordance with previous findings [61,64], and conflicting with others [68] that show that dairy and meat consumption were not significantly related to vitamin B12 status in a sample of 603 subjects, mean age 76.5 [68].

All in all, inadequate dietary intake is more likely to result in subclinical deficiency, revealed by biochemical markers rather than in clinical manifestation [45]. If the original B12 reserves (2–3 mg) were sufficiently large, dietary deficiency would deplete such store over several years [69].

## 4. Sustainable Strategies to Prevent Cbl Deficiency

Inadequate intake of B12 is frequently linked with sub-clinical B12 deficiency [45], with the exception of severe clinical abnormalities that should be treated intensively with B12 supplementation (oral, nasal, sublingual or intramuscular) [45]. In mild cases of B12 insufficiency, a primary prevention seems to be essential. Despite this review addressing B12 deficiency, it is important to underline that B12 works in synergy with several other micronutrients, primarily folate, but also vitamin B6 (as pyridoxal-5′-phosphate), riboflavin (as FAD), in one-carbon metabolism [2]. B-vitamins deficiencies may affect the complex regulatory network preserving one-carbon metabolism, despite adequate intake of vitamin B12 [20]. Therefore, considering public health issues related to B12 deficiency, a balanced approach must aim to achieve optimal status of all relevant B-vitamins [20].

Hereafter, the authors assess possible sustainable interventions that can be applied to prevent B12 deficiency in the elderly.

### 4.1. Food Fortification

The challenges and opportunities in achieving optimal B12 status in the elderly cover, first of all, food fortification (mandatory and/or voluntary). Mandatory fortification is that which is required by authorities (e.g., vitamin D fortification of milk or folic acid flour fortification) while voluntarily fortification is regulated by the manufacturer aiming at improving nutrient profile of food items (e.g., addition of vitamins and minerals to ready-to-eat breakfast cereals) [70]. 

Fortification is a tool that has been used successfully to correct nutrient inadequacies and their associated deficiencies. In the last years attention in fortification has interestingly shifted from prevention of deficiencies to improving health [71].

The experience of fortification has been successfully conducted for zinc, iron, selenium, vitamin A, vitamin B complexes (e.g., folate, niacin, thiamine), vitamin C, vitamin D and vitamin E [72] enrichment of foods. It is now well accepted that micronutrient fortification of foods has the potential to significantly increase serum micronutrient concentrations, reduce the incidence of micronutrient deficiencies in public health (e.g., Pellagra, Beriberi, childhood rickets, xerophthalmia), reduce clinical manifestations of deficiencies (including goiter, anemia and neural tube defects), and improve overall nutritional status [73,74].

There has been little experience with vitamin B12 fortification, especially on a large scale [63]. The most well-designed and documented national vitamin B12 fortification program took place in Cameroon, targeting fertile women and children [75]. The aim of this program was to follow the WHO/FAO guidelines on Food Fortification with Micronutrients [76] by assessing the micronutrient status of representative population groups (women aged 15–49 years and preschool children aged 12–59 months) and collecting data on dietary intake and patterns of consumption of specific foods and micronutrients before implementing a food fortification program [75].

In a small efficacy case-control trial in The Netherlands, healthy elderly who had not received B-vitamin supplements in the past 3 months nor vitamin B12 injections in the past 5 years and had not taken medication that interfered with folate or vitamin B12 metabolism (e.g., antacids) were provided with bread fortified with 9.6 μg B12/day as cyanocobalamin (cases) or unfortified bread (controls), for 12 weeks [77]. Results showed increased serum B12 in 49% cases and no B12 deficiency in anyone after fortification compared to about 8% in the initial sample, revealing also that B12 “survives” when added to flour and baked [77].

A randomized, double-blind trial study on 89 volunteers aged 50–85 years with no history of digestive or cardiovascular disease, uncontrolled hypertension, anemia, asthma, or cancer, regular use of multivitamin or B-vitamin supplements, medications that might interfere with B-vitamin metabolism, habitual consumption of highly fortified breakfast cereal or other food products fortified with B-vitamins, showed that one cup (0.24 L) of breakfast cereals fortified with folic acid (440 μg/cup), B12 (4.8 μg/cup) and B6 (1.8 mg/cup) increased blood concentrations of those vitamins and decreased plasma Hcy concentrations in the treatment group compared to the placebo one [64]. 

Effects of B12 supplementation have been studied using a milk product (containing cyanocobalamin = 7000 μg/L) administered to elderly individuals (n = 112, age ≥70) [78]. Only elderly with mild Cbl deficiency were enrolled (plasma Cbl between 100 and 300 pmol/L and a plasma MMA 0.30 ≥ μmol/L), while subjects with history of cobalamin (>50 μg/day) or folate (<200 μg/day) supplementation or injections, gastrointestinal surgery, renal dysfunction, anemia or cancer were excluded. The authors observed an increase in serum B12 levels, a decrease in MMA and in Hcy in the fortified-milk group [78]. All modifications were significantly different from those in the placebo group [78].

The data about a massive vitamin B12 fortification still remain scarce. Fortification policies should be suggested, considering fortification of foods poor in B12, preferably with multiple micronutrients [72].

Policy for B12 fortification remains controversial probably because effectiveness of vitamin B12 fortification critically depends on several physiological factors involving Cbl metabolism [2], suitable food vehicles [79], quality assurance and control programs [80].

Choosing an appropriate food vehicle to carry added nutrients is key to a successful fortification program [76]. It should be consumed in steady daily amounts, convenient for those at risk with no negative impact on palatability and added nutrients bioavailability [71]. 

As previously mentioned, food vehicles that have been employed successfully in B12 fortification program, specifically designed for the elderly, include bread, milk and breakfast cereals [64,77,78]; however, wheat flour, mineral water, nutrient bars and energy drinks have also been used, though in different target population (e.g., pregnant woman, children, adults <65 years) [77,78,81].

Lastly, all the factors that affect the design and implementation of food fortification policies must be considered in light of food technology limitations and reasonable cost.

Collaboration between food processing industry, government agencies and researchers is essential for the success of any food fortification program [71].

### 4.2. Novel Bacteria and In Situ Fortification 

As mentioned before, B12 synthesis appears to be restricted solely to some bacteria and archaea through two alternative routes: the aerobic or anaerobic pathway, respectively [82]. Some strains can also synthesize B12 by absorbing corrinoids via a salvage pathway as reviewed by Fang. H. et al. [83]. *Aerobacter*, *Agrobacterium*, *Alcaligenes*, *Azotobacter*, *Bacillus*, *Clostridium*, *Corynebacterium*, *Flavobacterium*, *Micromonospora*, *Mycobacterium*, *Norcardia*, *Propionibacterium*, *Protaminobacter*, *Proteus*, *Pseudomonas*, *Rhizobium*, *Salmonella*, *Serratia*, *Streptomyces*, *Streptococcus* and *Xanthomonas* are examples of B12-producing species [84]. The natural capacity for B12 production by certain bacteria has the potential to be exploited. B12 is not synthesized chemically by harnessing this natural capacity and enhancing it for in situ fortification of fermented foods [85]. 

Industrial vitamin B12 production employs microbial fermentation processes [85]. To date, numerous studies have been conducted regarding the use of the bacteria from Propionibacterium genus [86], specifically *P. denitrificans* and *P. freudenreichii* [82]. The major advantage of bacteria from the Propionibacterium genus is that they have the capacity to grow and synthesize metabolites on substrates containing different industrial waste products, which considerably elevates the economic profitability of biotechnological processes [87]. 

Other organisms producing vitamin B12, classified as GRAS (generally recognized as safe) species, are well known, yet due to different causes they are less attractive for the industry than Propionibacterium species. For instance, one of the most promising vitamin B12 producer, holding wide nutritional and health benefits, is the probiotic *Lactobacillus reuteri* [88]. Taranto stated that *L. reuteri* CRL1098, which belongs to lactic acid bacteria (LAB) and possesses a GRAS status, can synthesize B12 [89]. Yet, the product turned out to be a pseudovitamin B12 (i.e., inactive vitamin or low active compound) [90]. 

Recently, other strains in the genus *Lactobacillus* have shown to produce B12-type compounds, including *Lactobacillus coryniformis* isolated from goat milk [91], *Lactobacillus plantarum* from kanjika or Japanese pickles [92,93], *Lactobacillus rossiae* from sourdoughs [94] and *Lactobacillus fermentum* CFR 2195 from breast-fed healthy infants’ fecal sample [95], but further research is needed to elucidate their role in active B12 production. The addition of vitamin B12-producing LABs into fermented foods could potentially have two advantages: on one hand increase vitamin B12 concentrations, and on the other hand exhibit healthy properties, since in the elderly there is a general decrease in diversity exhibition in species of *Lactobacilli* [96]. 

In the field of food technology, mutation approaches and metabolic engineering have also proved to enhance B12 production by bacteria [97]. Recently, metabolic engineering has allowed overexpressing the genes involved in the biosynthesis of B12, but also via overexpressing genes involved in the biosynthesis of the target compound and related metabolites [98].

The concept of bacterial fermentation opens the way for development of food products targeted at specific groups in society such as the elderly [85], but more in vitro and in vivo studies are needed to test potential benefits health benefits. They potentially could place side by side fortification programs [97].

Moreover, the identification and characterization of novel bacteria with high B12 production may play crucial roles in preventing B12 deficiency [98].

### 4.3. Biofortification

Biofortification, of staple crop products, is a complementary food-based intervention [99]. It was ranked as the fifth most important strategy to improve global health by the Copenhagen Consensus [100], with a public health impact not only confined to rural areas and poor countries [101]. 

Most biofortification efforts to date have focused on vitamin A, iron and zinc, and their impact on human health. Yet, improved B-vitamin content may provide benefits to the human health [57]. 

Mozafar demonstrated that the addition of an organic fertilizer, specifically cow manure, significantly increases vitamin B12 content in spinach leaves and barley kernels, respectively [102]. Moreover, Mozafar and Oeftli investigated the uptake of B12 by soybean roots under water culture conditions, reporting after a five-day uptake period, a linear relationship between the concentration of vitamin B12 in the nutrient medium and in the leaves of soybean plants whose roots were placed into solutions containing non-labelled Cbl [103]. Sato et al. reported that a high level of vitamin B12 was incorporated into a vegetable, kaiware daikon (radish sprout), by soaking its seeds in B12 solutions before germination [104].

Despite the promising data, to the best of our knowledge no efficacy trials have been conducted to assess the value of biofortified crops bred with enhanced B12 vitamin content aimed at improving nutritional status in the elderly. 

Lastly and ideally, biofortified crops should not contain any variation in their texture, flavor or appearance [70]. If not, the new sensory characteristics have to be favorably assessed [105].

### 4.4. Vegetable Sources of Vitamin B12

The necessity to study and to find alternative sources of B12 among plant-origin foods arises from the demand to promote healthy dietary patterns [25].

Although some plant foods might serve as a source of B12 [106], literature still debates whether it is found in the active form, and whether regular consumption of these foods can be sustainable for the elderly [50]. Several edible mushrooms contain traces of B12 (e.g., porcini (*Boletus* sp.) and parasol mushrooms (*Macrolepiota procera)* [107]). However, an Italian study has shown that selected types of oyster mushrooms (*Pleurotus*) grown in the mountain areas in southern of Italy, specifically in Sicily, have a wide range of B12 from 0.44 to 1.93 μg/100 g [108]. Less common mushrooms such as *Craterellus cornucopioides* and *Cantharellus cibarius* may contain 1.09–2.65 μg/100 g [109]. Best known Asiatic Shiitake mushrooms (*Lentinula edodes*) can contain up to 5.61 ± 3.9 μg of Cbl per 100 g of dry weight (mostly in active form), although with great variability [110]. For instance, a portion of 50 g of dried shiitake could be adequate to achieve the daily requirement, thought this scenario is an unlikely everyday scenario [107,110]. 

Among edible algae, dried green (*Enteromorpha* sp.) and purple (*Porphyra* sp.) lavers (also known as nori) contain reasonable amounts of B12 ranging from 32 to 78 μg/100 g dry weight [111]. In vitro tests are promising, but there are not enough human clinical trials to consider the use of seaweed in supporting vitamin B12 requirements [112,113,114]. 

Several edible cyanobacteria, such as Spirulina, Aphanizomenon and Nostoc, contain significant amounts of corrinoids, many of which appear to be pseudovitamins [115], with poor nutritional value and unusable for humans [116]. At present, cyanobacteria cannot be considered a reliable source of vitamin B12 [116]. 

Although vegetable sources of B12 might represent an alternative and sustainable source of B12, it is unlikely that their daily use will represent a stable supply of B12 in the elderly [50]. This is even more true in the western countries where those foods are less consumed than in the eastern regions of the world [107]. More probably they can become a functional source of B12 to fill human requirements throughout food complements or nutraceuticals [106,115].

### 4.5. Supplementation

Substantial evidence supports supplementation programs as effective tools to optimize human health [117]. Regular use of dietary supplements was found to compensate (to some extent) nutritional inadequacies in the elderly, thereby filling the gap between recommended and actual intakes of micronutrients in this population [118]. Those factors made vertical programs (i.e., supplementation programs) attractive, not least because of their cost-effectiveness, also referring to B12 [119]. 

As already mentioned in this paper, vitamin B12 supplementation in pharmacological doses is an essential treatment for some people with clinical, symptomatic and severe deficiency, that could not possibly be reverted by a somewhat increased dietary intake of B12 [2]. Such cases require a prompt correction with very high doses of B12 [120,121], bypassing the specific uptake via intrinsic factor, necessary to bind B12 and mediate its endocytosis in the terminal ileum [4]. 

For a wider group of aging adults, dietary supplements may prevent or alleviate vitamin B12 deficiency if it is not caused by a severe malfunctioning of the specific uptake [4]. 

Examples of low-severity cases might cover: (i) chronic Helicobacter pylori infection, a common disorder in aged people [122] leading to type B gastritis, (ii) drugs interactions (e.g., Metformin, gastric acid suppression medications (H2RA, PPIs), anesthetic gas (NO2), Cholestyramine, Acetylsalicylic acid, Colchicine) [20,27], (iii) type A chronic atrophic gastritis [2], (iv) inflammatory bowel disease (Crohn disease and ulcerative colitis) specifically during the active phase of pathology [123], and (v) gastrointestinal surgery (partial or total gastrectomy including bariatric surgery; ileum resection) [45]. 

Finally, those on strict vegetarian and/or vegan diets should be recommended to supplement their diets with a reliable source of vitamin B12 [124]. 

B12 supplementation is known for its role in the prevention of several chronic age-related diseases, including cardiovascular and cerebrovascular diseases [125], cognitive impairment and mood disorders [125,126], loss of physical performance impairment and sarcopenia [127,128,129] and cancer risk [130].

B12 can be administered orally and parenterally (intramuscularly). Other methods include subcutaneous, transdermal, sublingual and nasal formulations [28], although their role in clinical practice appears marginal, because of their variable effectiveness and higher costs [131]. As reported in a Cochrane review [27], oral vitamin B12 or vitamin B12 administered intramuscularly might have similar effects in normalizing B12 serum levels, but oral treatment costs less [27]. This is very useful, as intramuscular administration is far more expensive and rather painful for the patient, as well as not free from complications [132]. Oral B12 supplementation represents an easy route of administration, more comfortable for end users and effective in mild-moderate deficiency [131]. Furthermore, it is also more appropriate in patients on anticoagulant treatment, in whom intramuscular injections may be contraindicated [28]. Despite all benefits, it should be mentioned that severe clinical deficiencies (e.g., neurological symptom, critically low B12 levels) should be treated aggressively with injections, to promptly provide a fast restoration of B12 stores [131]. Subsequently, patients may be able to convert to oral replacement with close monitoring [133].

Four formulations of B12 are commercially available [134]. To date, among all, cyanocobalamin and hydroxocobalamin are the most used [27], and in some countries hydroxocobalamin has completely replaced cyanocobalamin as first choice for vitamin B12 supplementation [135]. Likewise, the physiological forms of cobalamin (e.g., adenosylcobalamin and methylcobalamin) recently emerged as alternative forms in supplements with different routes of administrations [134].

Vitamin B12 can also be found in multi-mineral and vitamin supplements (MMV) [136], which often contain low-dose B12 (>5 μg/day) in association with other vitamins and minerals. They are mainly used to compensate poor nutrition during periods of fatigue, convalescence and recovery [27]. However, there is currently no evidence of their efficacy in preventing vitamin B12 deficiency [27] and some concerns deal with the ideal mix of vitamins and minerals in order to avoid processes of mutual inactivation. Indeed, an appreciable loss of B12 also occurs in B12-containing multivitamin supplements. Vitamin C does not degrade B12 per se, but in presence of copper leads to significant degradation of B12 and development of inactive products [137]. These compounds can inhibit the transport system interacting with transporter proteins [137]. 

Therefore, for an effective supplementation, biomarker assessment is crucial in order to establish type, dosage and timing of the supplementation. Identifying the cause of deficiency, such as inadequate intake, malabsorption, drug nutrients interactions, pathological cellular B12 uptake [138], or SF disorders [139] is critical, since not all them can be corrected by oral supplementation, indeed not even completely by intramuscular injections, which is the way vitamin B12 is traditionally administrated in clinical settings to avoid the frequent rate of low resorption due to the underlying disease [140].

Periodic assessment of B12 status is recommended [32]; however, total serum B12 is not the best biomarker for real B12 status assessment. Indeed, high total plasma B12 level can be observed together with a functional B12-deficiency [140].

B12-related metabolites (serum holoTC, serum Hcy and MMA levels) are much more reliable markers of the biochemical response [4]. Holotranscobalamin (HoloTC) is considered a better biomarker for early changes in B12 status than serum cobalamin concentration [4], although it can be affected by inborn errors altering intracellular vitamin B12 metabolism [141]. On the other hand, Homocysteine (Hcy) plasma concentration is affected by many confounding factors especially in the elderly, including folate deficiency, B6 deficiency, sarcopenia and renal impairment [2,4,28,142]. Methylmalonic acid (MMA), is considered the most specific and sensitive measure of B12 status, yet such analysis is more costly and this reduces its clinical availability [2]. Finally, in order to reach effective results following supplementation, it is necessary to continue it in the long-term, increasing direct costs for individuals more than adhering to a food fortification program [70].

## 5. Conclusions

A reduction of animal-source foods in diet is becoming more popular in western societies due to ethical, environmental, economic and health reasons, posing concerns about the beneficial or detrimental outcomes of these restrictions [50]. It is undoubtful that a dietary pattern rich in plant-based foods and poor in animal sources might benefit health and environment [22], but on the other hand it might lead to an inadequate intake of most notably vitamin B12 [2]. 

Such situation demands identification of sustainable sources of B12, as an alternative to exclusive meat consumption. This particularly concerns high-risk groups such as aging adults, for whom many factors apart from health concerns may contribute to the exclusion of animal source foods e.g., food pricing, age related constraints in chewing and/or swallowing, food access, as well as religious and cultural factors [20].

Given all the previous concerns, it is necessary to identify plant-source foods that naturally contain high levels of bioactive vitamin B12. Alternatively, one may consider fortification of foods [72]. 

Biofortification (adding vitamins and minerals to crops through plant biotechnology) is a promising approach for improving the nutritional status of a population. Moreover, the concept of in situ fortification by bacterial fermentation opens the way for innovative food products. Such strategies could be easily adopted by the food industry to develop novel vitamin B12-enhanced functional foods [85]. A long known multi-vitamin supplementation should be considered as a valid preventive treatment (even if it cannot provide the over-all long-term benefits that food-based approaches can deliver [70]). All together, these approaches would contribute to efficient measures to prevent general malnutrition in the elderly.

Global food policies involving multiple stakeholders and public–private partnership are required to help effective public health intervention to counteract expanding vitamin B12 deficiency.
